# Four new species of *Unixenus* Jones, 1944 (Diplopoda, Penicillata, Polyxenida) from Australia

**DOI:** 10.3897/zookeys.278.4765

**Published:** 2013-03-21

**Authors:** Megan Short, Cuong Huynh

**Affiliations:** 1 Deakin University, 221 Burwood Highway, Burwood, Melbourne, Australia

**Keywords:** Diplopoda, Polyxenida, Polyxenidae, *Unixenus*, millipede, Australia

## Abstract

*Unixenus carnarvonensis* **sp. n.**, *Unixenus corringlensis* **sp. n.**, *Unixenus barrabaensis* **sp. n.** and *Unixenus myallensis* **sp. n.** are described from Australia. A revised diagnosis of *Unixenus karajinensis* and new details on the distribution of the species are given. A key is presented to 10 of all 11 currently known species of the genus.

## Introduction

*Unixenus* Jones, 1944 is a widespread genus in the family Polyxenidae in the single order Polyxenida within the millipede subclass Penicillata. The genus is characterised by the presence of a single generalised type I caudal bundle of trichomes (Condé and Nguyen Duy-Jacquemin 2008) with a linear arrangement of ornamental trichomes above, and antennal articles VII with 2 thick basiconic sensilla, 1 setiform sensillum between, and 1 coeloconic sensillum posteriorly. Barbate trichomes are arranged posteriorly on each tergite in 2 or more rows with trichomes arranged in 2 broad clusters laterally. Tarsus 2 has one small seta.

Seven species have been described in *Unixenus*. The type species *Unixenus padmanabhii* (Jones, 1937) from India, *Unixenus broelemanni* (Condé & Jacquemin 1962) from Madagascar, *Unixenus vuillaumei* (Condé & Terver, 1963) from Ivory Coastand four Australian species *U. mjoebergi* (Verhoeff, 1924), *Unixenus attemsi* Nguyen Duy-Jacquemin & Condé, 1967, *Unixenus karajinenis* Short & Huynh, 2011 and *Unixenus corticolus* Short & Huynh, 2011. In this paper, four new species of *Unixenus* from Australiaare described. A revised diagnosis together with an expanded distribution is given for the recently described species *Unixenus karajinensis*, previously identified from just three locations in the Hamersley Ranges in the Pilbara, WA.

## Methods

The specimens in this study came from the collections of the Australian Museum in Sydney, NSW and the Western Australian museum, Perth, WA. Specimens were examined using light microscopy. For light microscopy, specimens were cleared in 15% potassium hydroxide, heated in a water-bath for 2 minutes at 80°C, neutralised in 20% acetic acid for 2 minutes, rinsed in distilled water and dehydrated in a series of ethanol baths prior to staining with 1% Fast Green solution to increase contrast. The head and body were separated, the body cut open with a single latero-longitudinal incision and contents removed. After rinsing in 100% ethanol, stained specimens were transferred to 100% isopropanol, then to xylene and mounted on slides with DPX synthetic resin.

Specimen lengths were measured from head to telson with the caudal bundle of trichomes excluded. Adults were sexed when possible. Naming of the leg segments follows Manton (1956). Unless otherwise indicated, all millipedes referred to are adults (stadium VIII). Stadium VII specimens are referred to as subadult, and “immature" refers to any non-adult stadium. The trichomes in a transverse row on the telson dorsal to the caudal bundle are referred to as ornamental trichomes.

Abbreviations: AM = Australian Museum, Sydney, New South Wales; NSW = New South Wales; WA = Western Australia; WAM = Western Australian Museum, Perth; L = left; R = right.

## Results

### Subclass Penicillata Latreille, 1831. Order Polyxenida Verhoeff, 1934. Superfamily Polyxenoidea Lucas, 1840. Family Polyxenidae Lucas, 1840

#### Unixenus

Jones, 1944

Monoxenus Jones, 1937: 138; Silvestri 1948: 216.
Unixenus Jones, 1944: 94; Nguyen Duy-Jacquemin and Condé 1967: 68.

##### Type species:

*Unixenus padmanabhii* (Jones, 1937).

#### Unixenus carnarvonensis

Short & Huynh sp. n.

urn:lsid:zoobank.org:act:2FCC1AEE-BC97-4DB1-BCD2-04208BBA2C0D

http://species-id.net/wiki/Unixenus_carnarvonensis

Fig. 1A–K

##### Holotype.

Female, Meedo Station, WA, site MO3, 25°39'13"S, 114°37'37"E, wet pitfall traps 22 August–11 October 1994, collected by P. West et al., WAM T71123. Specimen mounted on slide, deposited in WAM.

##### Paratypes.

One male and 2 females, slightly damaged, Woodleigh Station, WA, site WO5, 26°11'45"S, 114°25'23"E, wet pitfall traps 22 August–10 October 1994, collected by M. Harvey et al., WAM T71120 (male), T127782 and T127783 (females). One female, same collection as holotype, WAM T127781. All paratypes mounted on slides, deposited in WAM.

##### Etymology.

For Carnarvon region, the type locality; adjective. All specimens of this species identified to date were collected as part of the Carnarvon Survey carried out by WAM and the Department of Conservation and Land Management (now part of Department of Environment and Conservation).

**Diagnosis.** Antennal article VI with 3 basiconic sensilla, 1 coeloconic sensillum, no associated setiform sensillum; seta present on tarsus 1 of legs 2–13, elongate smooth sided setiform setae on leg segments, ornamental trichomes *b* with large insertion points.

##### Description.

Measurements: Body length 2.8–3.3 mm with no differences between sexes, caudal bundle 0.3 mm.

No freshly collected specimens available. Specimens had been preserved in 70% ethanol. Body yellow brown in colour, trichomes largely missing.

**Figure 1.**
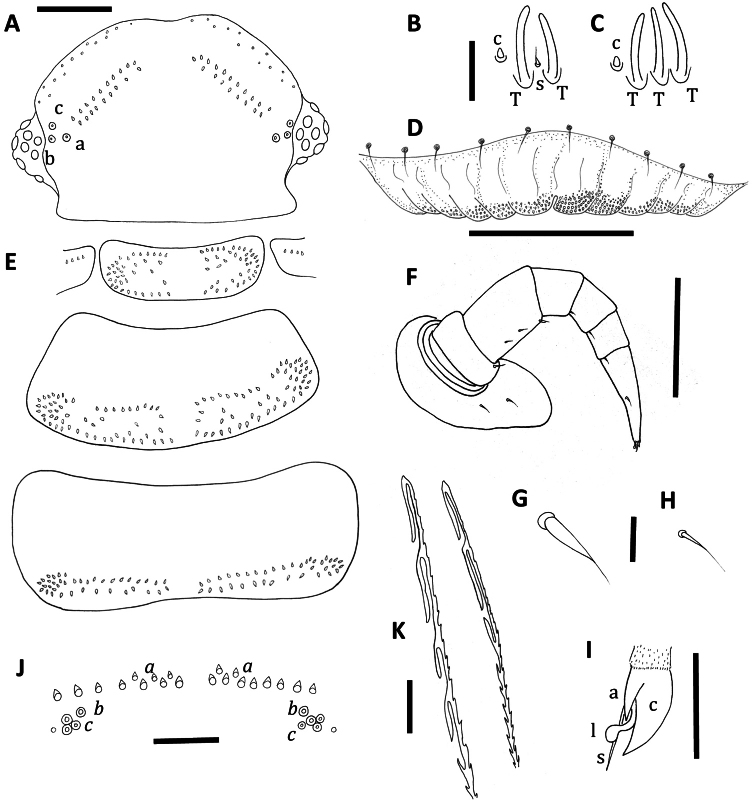
*Unixenus carnarvonensis* sp. n., adult female. **A** Head, dorsal view showing arrangement of ocelli, position of trichobothria (a, b and c) and trichome insertions **B** Details of sensilla on antennal article VII, sensillum type indicated as follows: coeloconic (**c**), setiform (**s**), thick basiconic (**T**) **C** Details of sensilla on antennal article VI **D** Clypeo-labrum **E** Collum, tergite 2 and tergite 10 showing pattern of trichome insertions **F** Left leg 3 showing chaetotaxy on leg segments **G** Details of seta on coxa, prefemur and femur **H** Details of seta on tarsus 2 **I** Anterior view of left telotarsus showing anterior spinous projection (**s**), claw (**c**) with anterior (**a**) process and lamella (**l**) **J** Pattern of ornamental trichome insertions **K** Detail of distal ends of hooked caudal trichomes, Scale bars: **A** and **E** (shared bar), **F** = 50 µm; **D** and **J** = 30 µm; **K** = 10 µm; **B** and **C** (shared bar) **G H, I** = 5 µm.

Head with 8 ocelli each side: 4 dorsal, 4 lateral (1 anterior, 2 medial and 1 posterior). Vertex with anterior rows of trichomes arranged as typical for the genus *Unixenus*, with a gap to two posterior groups of trichomes arranged in 2 closely positioned straight oblique rows. Number of trichomes in posterior rows of vertex of head each side varies from 10–13 (anterior), 5–9 (posterior). Trichobothria equal in size, arranged in shape of isosceles triangle with greater width a–c (Fig. 1A).

The antennae with proportions of 8 articles and 4 sensitive cones typical of the genus. Article VI with 3 thick basiconic sensilla of equal length, coeloconic sensillum posterior to basiconic sensilla (Fig. 1C). Antennal segment VII typical of the genus with 1 coeloconic sensillum to the posterior followed anteriorly by 2 thick basiconic sensilla of similar height; 1 setiform sensillum between the basiconic sensilla (Fig. 1B). Clypeo-labrum with 10 setae along posterior margin; anterior margin of labrum with 5–6 rounded lamellar teeth each side of median cleft; surface covered with tiny spherical papillae, papillae reducing in size to the posterior margin and lacking tiny hairs (Fig. 1D). Gnathochilarium typical of the genus with lateral palp 2.5 × length of medial palp; lateral palp with 13–15 cylindrical sensilla, medial palp with 22 sensilla.

Collum with arrangement of trichomes on tergites similar to *Unixenus mjoebergi*, with symmetrical pattern of trichomes each side of a broad median gap, with anterior and posterior rows merging laterally to form rosettes of trichomes, and further trichomes scattered between these rows. Small lateral protuberances each with row of 4–6 forward facing trichomes (Fig. 1E). Tergites 2–9 with trichomes arranged on posterior half of the tergite with one distinct posterior row with a medial gap and ending with small clusters laterally. Further trichomes anterior to this row loosely arranged in two to three rows (Fig. 1E). Anterior trichomes directed towards head while remaining trichomes directed posteriorly. Tergite 10 with 2 rows of trichomes arranged along posterior edge with broad medial gap (Fig. 1E). Conical pleural projections along each side associated with tergites 2–10, each with a dense cluster of trichomes. Tergal trichomes barbate, same structure as *Unixenus mjoebergi*.

Legs 1 and 2 without trochanter, leg 1 also lacks tarsus 1. Trochanters legs 3–13 lack setae. Chaetotaxy as follows: coxa 1, 1 seta, coxa 2, 2 setae, coxae 3–13, 2–3 setae; prefemur, postfemur, tarsus 2, legs 1–13 and tarsus 1, legs 2–13, with 1 seta; femur 1, 1 seta, femur 2, 1–2 setae, femur 3–13, 2–3 setae (Fig. 1F), setae distinctive elongate setiform (Fig. 1G) with distal seta of femur longer, small setae on postfemur, tibia, tarsus 1 and tarsus 2 (Fig. 1H); telotarsus bearing slender anterior spinous projection longer than the claw which bears anterior process, presence of posterior process unable to be determined, lamella process present (Fig. 1I). Male coxal glands not visible due to damage.

Telson with ornamental trichome insertions arranged almost symmetrically with 6–10 trichomes *a*, 1*b*, and 3*c* each side of the midline (holotype has 4 trichomes *c* on right side). Large insertion points for trichomes *b* (Fig. 1J). A small indentation external to trichomes *c*, either side. Single caudal bundle typical of genus. Hooked caudal trichomes with 2–5 hooks on barbed stems, majority with distal facing barbs only along stem proximal to hooks (Fig. 1K), some with distal and proximal facing barbs.

##### Distribution.

So far known only from two sites in the Carnarvon region of Western Australia (Fig. 5). Co-occurs with *Unixenus mjoebergi*.

**Remarks.** This species is very similar to *Unixenus mjoebergi* and cannot be separated without examination under high magnification.

#### Unixenus corringlensis

Short & Huynh sp. n.

urn:lsid:zoobank.org:act:FEA7C5BF-F58F-4DF4-8A25-506C6354C0B5

http://species-id.net/wiki/Unixenus_corringlensis

Fig. 2A–I

##### Holotype.

Female, Corringle State Forest, NSW, 33°22'12"S, 147°15'00"E, 21–26 March 1996, collected by D. Smith, AM KS.119541. Specimen mounted on slides, deposited in AM.

##### Paratypes.

1 male, same collection as holotype, AM KS.119540. 1 female and 1 male subadult stadium VII, Severn State Forest, Atholwood Loop Rd. 29°04'28"S, 151°00'53"E, wet pitfall traps 22 November–13 December 2001, collected by L. Wilkie and H. Smith, AM KS.87406 (male), AM KS.119542 (female). 1 female adult, Newnes State Forest, Birds Rock Flora Reserve, 0.6 km from Sunnyside Ridge Rd., 33°19'43"S, 150°11'33"E, litter sampling 23 Feb 2006, collected by G.A. Milledge, J. Tarnawski and M. Beatson. AM KS.119543. All paratypes mounted on slides and deposited in AM.

##### Other material:

4 specimens (sex and stadia not determined), Severn State Forest, Atholwood Loop Rd. 29°04'28"S, 151°00'53"E, wet pitfall traps 22 November–13 December 2001, collected by L. Wilkie and H. Smith. AM KS.87400, KS.87410, preserved in ethanol. 2 immature specimens, (sex and stadia not determined), Corringle State Forest, NSW, 33°22'12"S, 147°15'00"E, 21–26 March 1996, collected by D. Smith, AM KS.53604, preserved in ethanol.

1 male stadium VII, Newnes State Forest, Birds Rock Flora Reserve, 0.6km from Sunnyside Ridge Rd., 33°19'43"S, 150°11'33"E, litter sampling 23 Feb 2006, collected by G.A. Milledge, J. Tarnawski and M. Beatson, AM KS.119544, mounted on slide, poor quality.

##### Etymology.

For Corringle State Forest, the type locality; adjective.

##### Diagnosis.

Similar to *Unixenus attemsi* with biarticulate setae on leg segments and proximal position of seta on tarsus 2. Differs from *Unixenus attemsi* in being larger, having 2 setae on femur and 1 seta on tibia, maximum of 3 setae on coxa, broad gap between anterior rows of trichomes on vertex and posterior rows, and in arrangement of posterior rows.

##### Description.

As for *Unixenus carnarvonensis* sp. n., differing in the following details:

Measurements: Body length 2.3–2.5 mm with no differences between sexes, caudal bundle 0.3 mm.

No freshly collected specimens available. Specimens had been preserved in 70% ethanol. Body yellow brown in colour with tergal trichomes medium brown.

**Figure 2.**
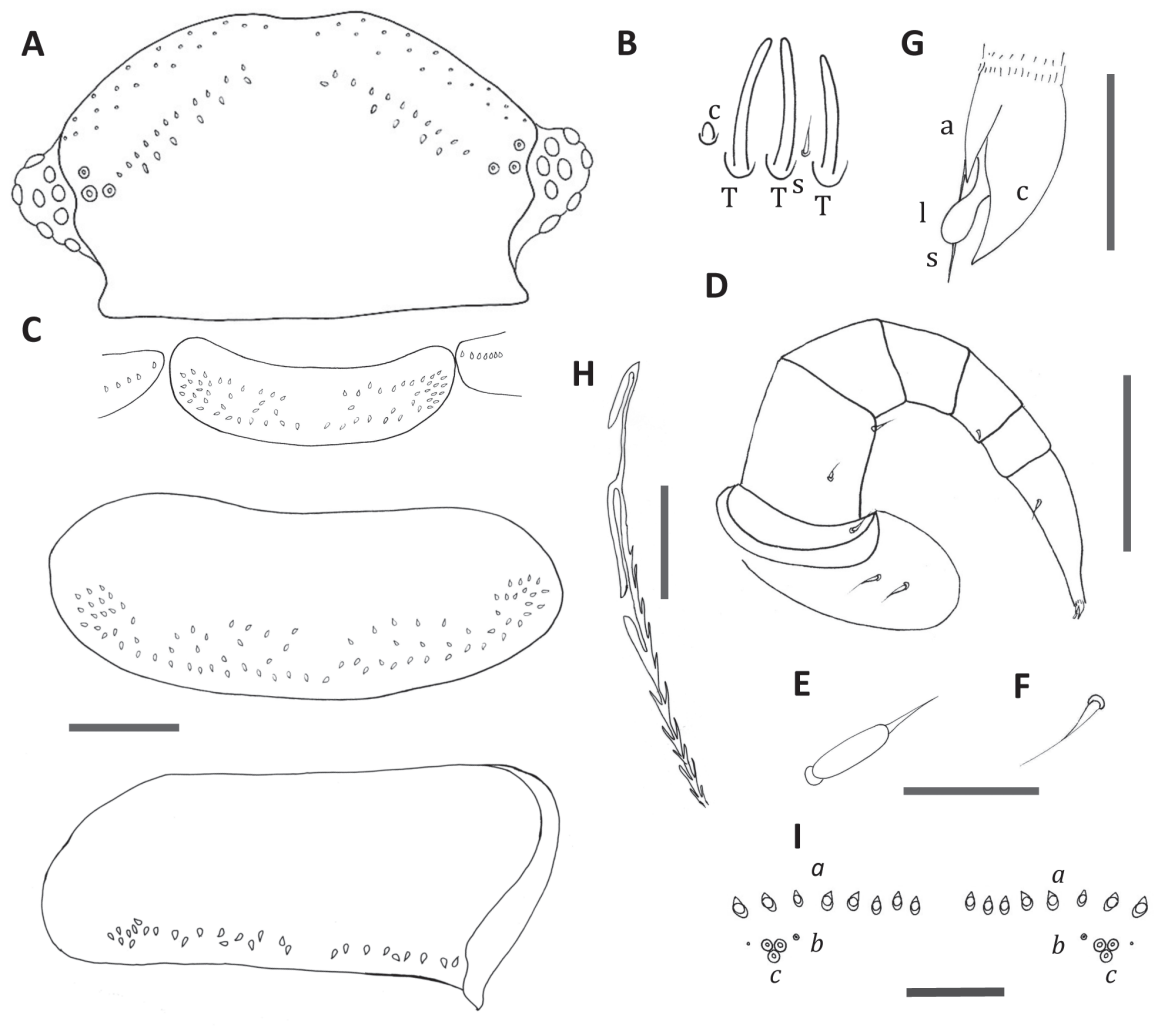
*Unixenus corringlensis* sp. n., adult female **A** Head, dorsal view showing arrangement of ocelli, position of trichobothria and trichome insertions **B** Details of sensilla on antennal article VI, sensillum type indicated as follows: coeloconic (**c**), setiform (**s**), thick basiconic (T) **C** Collum and tergite 2 (holotype), tergite 10 (paratype male) showing pattern of trichome insertions. Tergite 10 pattern partially obscured due to folding **D** Leg 3 showing chaetotaxy **E** Details of seta of coxa, prefemur and femur **F** Details of seta on tarsus 2 **G** Anterior view of telotarsus showing anterior spinous projection (**s**), claw (**c**) with anterior (**a**) process and lamella (l) **H** Hooked caudal trichome, distal section only **I** Pattern of ornamental trichome insertions. Scale bars: **A** and **C** (shared bar), **D** = 50 µm **B,** **E** and **F** (shared bar), **G** = 5 µm; **H** and I = 20 µm.

Number of trichomes in posterior rows of vertex of head each side varies 10–12 (anterior rows), 4–6 (posterior rows) (Fig. 2A). Antennal article VI with 3 thick basiconic sensilla, anterior sensillum shorter, coeloconic sensillum posterior to basiconic sensilla, setiform sensillum between basiconic sensilla 1 and 2 (Fig. 2B). Clypeo-labrum similar to *Unixenus carnarvonensis* sp. n. with 10 setae along posterior margin and fine sandy granular surface, granules decreasing in size to the posterior. Differs from *Unixenus carnarvonensis* sp. n. in having only 4 overlapping lamellar teeth each side of the median cleft. Lateral palp of gnathochilarium with 13 cylindrical sensilla, medial palp 20–21 sensilla.

Lateral protuberances of collum with 5–7 trichomes each. Tergites 2–9 with presence of median gap variable and narrow if present. Tergite 10 with median gap and reduced number of trichomes (Fig. 2C). Trichomes short barbate. Legs with similar chaetotaxy to *Unixenus mjoebergi* with coxa 1, 1 seta, coxa 2, 2 setae, coxae 3–13, 2–3 setae; prefemur, tibia and tarsus 2, 1 seta; femur, 2 setae; trochanter, postfemur and tarsus 1 lack setae (Fig. 2D). Setae same as for *Unixenus attemsi*, biarticulate with base lacking ridges and spines (Fig. 2E), seta on distal edge of femur with longer flagellum; setiform, tarsus 2 seta slightly longer and located more proximal than in *Unixenus mjoebergi* (Fig. 2F); telotarsus bearing anterior spinous projection longer than the wide claw which bears anterior process, posterior process not visible, small thin lamella process (Fig. 2G). Last sternal plate with 2 setae. Male with 2 pairs coxal glands on leg pairs 8 and 9.

Telson with ornamental trichome insertions numbering 7–10*a*, 1*b*, and 3*c* each side of the midline (Fig. 2I). Trichome *b* insertion points small, typical of the genus. Hooked caudal trichomes with 2–4 hooks on barbed stems, distal facing barbs along stem proximal to hooks, most distal barb larger. Caudal trichomes with 2 hooks only in both holotype and paratype from Corringle State Forest (Fig. 2H).

##### Distribution.

So far known only from State forest at two widely separated sites in mid NSW and one site in northern NSW (Fig. 5).

#### Unixenus barrabaensis

Short & Huynh sp. n.

urn:lsid:zoobank.org:act:7DED37AD-453D-4C20-BF47-FC344D23A958

http://species-id.net/wiki/Unixenus_barrabaensis

Fig. 3A–I

##### Holotype.

Female, Crown Reserve, Woods Reef, between road and Nangahrah Ck. NSW, 30°23'39"S, 150°44'08"E, pitfall traps 18 November–9 December 2001, collected by H. Doherty and M. Elliot, AM KS.119545. Specimen mounted on slide, deposited in AM.

##### Paratypes.

Male subadult stadium VII, same collection as holotype, AM KS.119546. Male subadult stadium VII Crown Reserve, 8.9 km along Bukkulla-Ashford Rd., NSW, 29°25'59"S, 151°04'18"E, pitfall traps 22 November–13 December 2001, collected by H. Doherty and M. Elliot, AM KS.119547. Female, stadium VII, Oaky Creek Nature Reserve, at base of E side of Melville Range, 31°06'31"S, 150°37'20"E, wet pitfall traps 17 November–8 December 2001, collected by L. Wilkie and H. Smith, AM KS.119548. Specimens mounted on slides, deposited in AM.

##### Etymology.

For Barraba, town closest to type locality; adjective.

##### Diagnosis.

Chaetotaxy similar to *Unixenus mjoebergi* with leg setae being spiny, 2 setae on femur and 1 on tibia; differs from *Unixenus mjoebergi* in having four basiconic sensilla on antennal article VI.

##### Description.

As for *Unixenus carnarvonensis* sp. n., differing in the following details:

Measurements: female adult, 1.94 mm, caudal bundle 0.3 mm; male subadult, Stadium VII, 1.6–2.1 mm. (n=3), caudal bundle 0.3 mm.

No freshly collected specimens available. Specimens had been preserved in 70% ethanol. Body yellow brown in colour with brown tergal trichomes.

**Figure 3.**
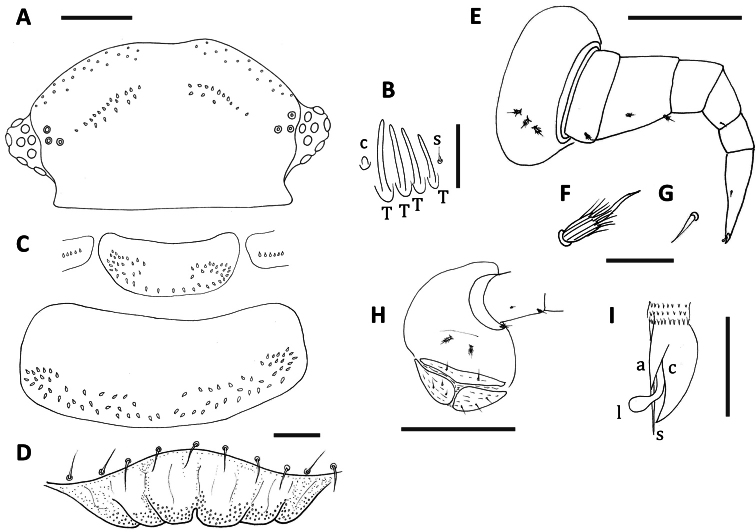
*Unixenus barrabaensis* sp. n., adult female **A** Head, dorsal view showing arrangement of ocelli, position of trichobothria and trichome insertions **B** Details of sensilla on antennal article VI, sensillum type indicated as follows: coeloconic (**c**), setiform (**s**), thick basiconic (**T**) **C** Collum and tergite 2 showing pattern of trichome insertions **D** Clypeo-labrum **E** Left leg 3 showing chaetotaxy on leg segments **F** Details of seta on coxa, prefemur and femur **G** Details of seta on tarsus 2 **H** Detail of coxa and vulva of left leg 2 **I** Anterior view of left telotarsus showing anterior spinous projection (**s**), claw (**c**) with anterior (**a**) process and lamella (**l**). Scale bars: **A** and **C** (shared bar), **E** and H = 50 µm; D = 10 µm **B** Fand G (shared bar) and I = 5 µm.

Two rows of trichomes either side of posterior vertex of head with slightly larger medial gap than for *Unixenus carnarvonensis* sp. n. Number of trichomes each side of posterior vertex varies 8–14 (anterior rows), 4–6 (posterior rows) (Fig. 3A). Antennal article VI with 4 thick basiconic sensilla, coeloconic sensillum posterior to basiconic sensilla, One setiform sensillum anterior to the basiconic sensilla (Fig. 3B). Clypeo-labrum with 10 setae along posterior margin in holotype, 8–10 in subadult paratypes; anterior margin of labrum with 3–4 lamellar teeth each side of the median cleft; fine sandy granular surface, granules becoming smaller posteriorly (Fig. 3D). Lateral palp of gnathochilarium typical of the genus with 13 cylindrical sensilla.

Collum and tergites 2–9 with small median gap in posterior row of trichomes (Fig. 3C). Rows increasingly closer together in posterior tergites. Tergite 10 of holotype damaged so size of median gap unable to be determined. Leg chaetotaxy as follows: coxa 1, 1 seta, coxa 2, 2 setae, coxae 3–13, 2–3 setae; prefemur, tibia, tarsus 2, 1 seta; femur, 2 setae (Fig. 3E). Setae of the coxa, prefemur and distal edge femur with biarticulate setae similar to those for *Unixenus mjoebergi* with longitudinal ridges on basal funicle, each ridge extending distally in a long, thin spine with the spines surrounding the base of the flagellum (Fig. 3F); seta of mid femur similar but smaller, tibia and tarsus 2 with setiform setae (Fig. 3G); telotarsus bearing anterior spinous projection longer than the claw which bears large anterior process ½ length of claw, no posterior process visible, short lamella process (Fig. 3I). Vulvae of adult female typical of genus, with numerous setae (Fig. 3H). Male with 2 pairs coxal glands on leg pairs 8 and 9.

Telson with ornamental trichome insertions numbering 6–8*a*, 1*b*, and 3*c* each side of the midline, arrangement typical for the genus, as illustrated for *Unixenus corringlensis*. These trichomes barbate, long and straight. Hooked caudal trichomes with 1–3 hooks on barbed stems, majority with distal facing barbs only along stem proximal to hooks, very small number with distal and proximal facing barbs.

##### Distribution.

So far known only from three forested sites in northern NSW, with a north-south range of ca 200 km (Fig. 5).

#### Unixenus myallensis

Short & Huynh sp. n.

urn:lsid:zoobank.org:act:1DB55177-48AA-48A3-AF88-4820D4706C7E

http://species-id.net/wiki/Unixenus_myallensis

Fig. 4A–K

##### Holotype.

Female, Myall Lakes National Park, 3.8 km South of Mungo Brush campsite, NSW, 32°34'46"S, 152°17'27"E, ca 300–500 m, wet pitfall traps 14–24 May 1998, collected by L. Wilkie, AM KS.93670. Specimen mounted on slide, deposited in AM.

##### Paratypes.

Male subadult stadium VII, same collection as holotype, AM KS.94096.

Female, same site as holotype, wet pitfall traps 26 November 1997, collected by L. Wilkie, AM KS.93660. Male adult, Male subadult stadium VII, Female subadult stadium VII, Wyrrabalong National Park, NSW, 33°16'47"S, 151°32'40"E, wet pitfall traps 27 November 1997, collected by L. Wilkie, AM KS.93662, KS.119556, AM KS.93663. All paratypes mounted on slides, deposited in AM.

##### Other material:

1 immature stadium IV (6 pairs legs), same site as holotype, wet pitfall traps 14 May 1998 collected by L. Wilkie, KS.93665. 1 stadium VI, 10 pl, sex unknown, Wyrrabalong National Park, NSW, 33°16'48"S, 151°32'45"E, wet pitfall traps 27 November 1997, collected by L. Wilkie, AM KS.93659. 1 stadium V, 8 pl, 1 stadium IV, 6 pl, Wyrrabalong National Park, NSW, 33°16'47"S, 151°32'40"E, wet pitfall traps 27 November 1997, collected by L. Wilkie, AM KS.93661, AM KS.93666. Preserved in ethanol in AM.

##### Etymology.

For Myall Lakes National Park, the type locality; adjective.

##### Diagnosis.

Similar chaetotaxy to *Unixenus attemsi* with 1 seta on femur and none on tibia, setae smooth biarticulate; differs from *Unixenus attemsi* in having a gap between anterior and posterior vertex groups of trichomes, 4 basiconic sensilla on antennal article VI, gnathochilarium with short lateral palps (1.5 × diameter of medial palp) bearing 12–13 short rounded sensilla, ornamental trichome insertions *c* in row.

##### Description.

As for *Unixenus carnarvonensis*, differing in the following details:

Measurements: Body length 2.2–2.4 mm (n=3) with no differences between sexes. Caudal bundle 0.5 mm.

Colouration: No freshly collected specimens available. Specimens had been preserved in 70% ethanol. Body yellow brown in colour with black trichomes including caudal bundle dark brown – black.

**Figure 4.**
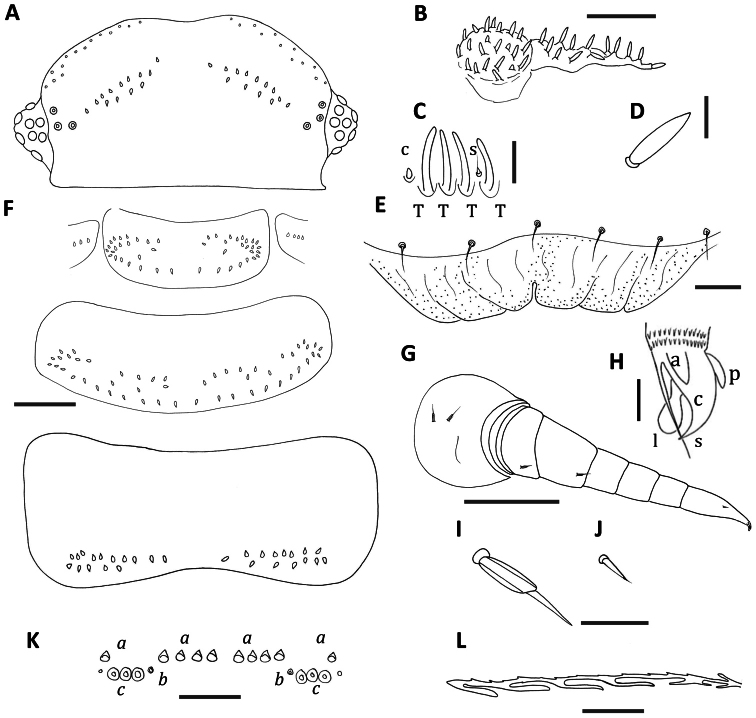
*Unixenus myallensis* sp. n., adult female **A** Head, dorsal view showing arrangement of ocelli, position of trichobothria and trichome insertions **B** Left palp of gnathochilarium **C** Details of sensilla on antennal article VI, sensillum type indicated as follows: coeloconic (**c**), setiform (**s**), thick basiconic (**T**) **D** Details of gnathochilarial sensillum **E** Clypeo-labrum **F** Collum, tergite 2 and tergite 10 showing pattern of trichome insertions **G** Left leg 3 showing chaetotaxy on leg segments **H** Anterior view of left telotarsus showing anterior spinous projection (**s**), claw (**c**) with anterior (**a**) and posterior (**p**) processes and lamella (**l**) **I** Details of seta on coxa, prefemur and femur **J** Details of seta on tarsus 2 **K** Pattern of ornamental trichome insertions **L** Hooked caudal trichome, distal section only. Scale bars: **A** and **F** (shared bar), **G** = 50 µm; **B** and **K** = 20 µm; **E** = 10 µm; **I** and **J** (shared bar) and **L** = 5 µm **C,** **D** and **H** = 2 µm.

Two rows of trichomes either side of posterior vertex of head with number of trichomes in rows varying from 7–11 trichomes each side anteriorly, 4–6 each side in posterior rows. (Fig. 4A). Antennal article VI with 4 thick basiconic sensilla, coeloconic sensillum posterior to the basiconic sensilla, setiform sensillum between basiconic sensilla 1 and 2 (Fig. 4C). Clypeo-labrum with 6–9 setae along the posterior margin; anterior margin of labrum with 3–5 square shaped lamellar teeth each side of median cleft; tiny granular structures over labrum (Fig. 4E).

Gnathochilarium with lateral palp 1.5 times length of medial palp. Lateral palp with 12–13 sensilla, medial palp 22 sensilla (Fig. 4B), sensilla fat, cylindrical and short (2/3 length of *Unixenus attemsi* sensilla) (Fig. 4D).

Collum with two rows of trichomes sparsely arranged each side of a medial gap, rows merge laterally to form rosettes of trichomes, and with a small number (2 each side in holotype) of trichomes between the two rows. Lateral protuberances of collum with 3–4 trichomes each. Tergites 2–10 with trichomes arranged most commonly in 2 rows on posterior half of the tergite each side of a medial gap. Anterior row sinuous with small gap to lateral cluster. In posterior tergites the two rows are closer together and straighter (Fig. 4F). In some specimens tergites 2 and 3 have scattered trichomes between the anterior and posterior rows forming an intermediate 3^rd^ row. Conical pleural projections along each side associated with tergites 2–10, each with dense cluster of short dark trichomes. Tergal trichomes of posterior tergites longer than those of anterior tergites.

Leg chaetotaxy as follows: coxa 1, one seta, coxa 2, 2 setae, coxae 3–13, 2–3 setae; prefemur, femur, 1 seta (Fig. 4G); setae biarticulate with glabrous funicle showing longitudinal ridging (Fig. 4I), tarsus 2 with small setiform seta (Fig. 4J), trochanter, postfemur, tibia and tarsus 1 lack setae, claw of telotarsus bearing posterior and anterior processes (Fig. 4H). Male with 2 pairs coxal glands on leg pairs 8 and 9.

Telson with ornamental trichome insertions numbering 5–6*a*, 1*b* and 3*c* each side of the midline, with *c* trichome insertions in straight line (Fig. 4K). Trichomes *b* and *c* black in colour. Hooked caudal trichomes with 1–6 hooks on barbed stems, majority with distal facing barbs only along stem proximal to hooks, with occasional double barbs with both distal and proximal facing projections (Fig. 4L).

**Figure 5.**
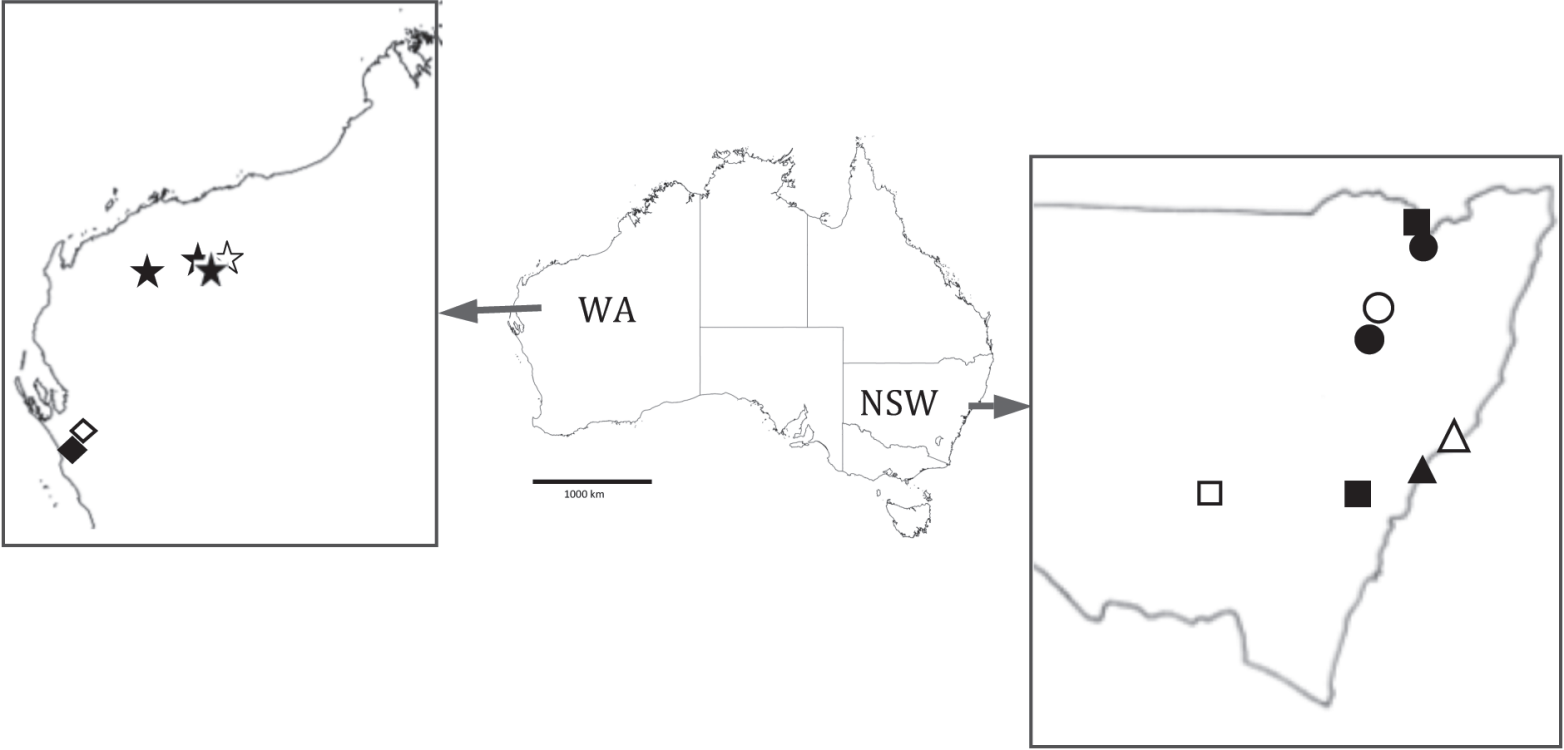
Map of Australia with states New South Wales and Western Australia expanded to show distribution of five *Unixenus* species In Western Australia: *Unixenus carnarvonensis* sp. n.: diamonds, type region shown as open diamond *Unixenus karajinensis* Short and Huynh 2011: stars, type locality shown as open star. In New South Wales: *Unixenus corringlensis* sp. n.: squares,squares, type locality shown as open square *Unixenus barrabaensis* sp. n.: circles, type locality shown as open circle *Unixenus myallensis* sp. n.: triangles, type locality shown as open triangle. Scale bar =1000 km.

**Distribution.** So far known only from two treed coastal sites in mid NSW, with a north-south range of ca 150 km (Fig. 5).

#### Unixenus karajinensis

Short & Huynh, 2011

http://species-id.net/wiki/Unixenus karajinensis\according to Short et al 2013

##### Material examined.

A number of collections of various stadia, in poor quality: Wittenoom WA, May 1973, EN Wahl, WAM T116466, T116467, T116469; Wittenoom, WA, from out of drain pipe in bath at night, also on floors in house, 10 October 1983, Mrs. M. McKay, WAM T116468; Tom Price Caravan park, Tom Price, WA, 22 December 1982, K. Campbell, WAM T116450; Tom Price Caravan park, Tom Price, WA, 2 December 1982, A. Davies, WAM T116470; Hamersley Station, WA, under house, 20 April, 1989, JS Bogle, T117557; Mt Stuart Station, 0.7 km W Urandy Bore (1: 100,000 map ref: 2153-283201), huge numbers on surface of ground (gibber slope) at night, 6 August 1985, large collection in very poor condition, A Baynes and TA Smith, WAM T117558. Selected specimens mounted on slides for identification, remainder in ethanol, all deposited in WAM.

##### Revised diagnosis.

Differs from *Unixenus mjoebergi* in longer and thinner tergal trichomes, 6 pairs of coxal glands in males on leg pairs 6–11, telotarsus with anterior spinous projection shorter than the claw, 5–9 ornamental trichomes *c* each side. Antennal articles VI and VII with distinctive notched appearance at the distal edge, article VI with setiform sensillum anterior to 3 basiconic sensilla. Number of setae on coxae 3–13 varies more widely from 1–6 in contrast to 2–3 in *Unixenus mjoebergi*. The hooked caudal trichomes have double barbs proximal to the hooks. The last sternal plate has 2 setae.

##### Remarks.

Examination of further specimens of *Unixenus karajinenis* has confirmed that the original diagnosis for the species is in error in stating that the number of ornamental trichomes *c* is 8 each side (Short and Huynh 2011). Although the number is 8 each side in the majority of specimens examined, the number can vary from 5–9.

A number of the collections examined were from the two previous collection sites: the type locality Wittenoom and the nearby township of Tom Price. However the species has now been identified from two further locations in the Pilbara region of Western Australia, with one site being 200 km from the type locality indicating that its distribution is not as tightly restricted to the Hamersley Ranges as originally recorded (Fig. 5).

Collections were sent to WAM for identification after reports from 1972 of the millipedes reaching nuisance proportions in parts of the Pilbara, particularly the Hamersley Ranges area and the townships of Tom Price and Wittenoom (Koch 1985). Koch identified the species involved as *Unixenus mjoebergi* and a study was done for the Western Australian Department of Agriculture (Burt 1984) to determine ways of reducing millipede numbers and swarming behaviour. However after recent examination of collections in WAM it appears that *Unixenus karajinensis* also occurred in huge numbers and has been found associated with housing. It appears very likely to have been involved in swarming behaviour.

## Discussion

The genus *Unixenus* is widespread and speciose. It is likely further species will be identified. The genus is not limited to Australia. Apart from *Unixenus padmanabhii* in India, *Unixenus vuillaumei* in Ivory Coast and *Unixenus broelemanni* in Madagascar, specimens from two locations in Vietnam have been identified as *Unixenus* by Nguyen Duy-Jacquemin and Condé (1967), as well as two specimens from Vietnam retrieved from imported tropical fruit by the Australian Quarantine and Inspection Service and identified by the authors (QM collection QMS 25102, prepared as slides) as being in the genus *Unixenus*. Specimens from 3 locations in Papua New Guinea have been identified as 3 distinct species of *Unixenus* (Nguyen Duy-Jacquemin and Condé 1982, Condé and Nguyen Duy-Jacquemin 1984). The specimens are tiny juveniles (stadia II and III) and were identified as *Unixenus*, most probably from 3 undescribed species. Adult or subadult specimens will be required for complete identification.

### Updated Key to described species of *Unixenus*

The type species from India, *Unixenus padmanabhii* is not included as insufficient details are known.

Unfortunately the species in the genus *Unixenus* are very similar and reliable characters suitable for identification are only visible under high magnification, requiring preparation of slides. The key should be used with some caution as although it has been developed using characters that are consistent in the individuals examined for each species, there may be some variability in characters that has not been discernable due to the limited number of adult individuals available for examination.

**Table:** 

1	Presence of setae without projecting spines on legs	2
–	Presence of setae with projecting spines on legs	6
2	One seta only on femur, no setae on tibia of legs 6–13	3
–	At least 2 setae on femur, 1 seta on tibia	5
3	3 basiconic sensilla on antennal article VI	4
–	4 basiconic sensilla on antennal article VI, to date found only in NSW, Australia	*Unixenus myallensis* sp. n.
4	Telotarsus with more than 2 processes on claw, to date found only in Australia	*Unixenus attemsi*
–	Telotarsus with 2 or less processes on claw, to date found only in Madagascar	*Unixenus broelemanni*
5	No seta on tarsus 1 of legs 3–13, small or no median gap in posterior row of trichomes on tergite 2, setiform sensillum between basiconic sensilla 1 and 2 on antennal article VI, to date found only in NSW, Australia	*Unixenus corringlensis* sp. n.
–	Seta present on tarsus 1 of legs 3–13, Median gap in posterior row of trichomes on tergite 2, no setiform sensillum between basiconic sensilla 1 and 2 on antennal article VI, to date found only in WA, Australia	*Unixenus carnarvonensis* sp. n
6	One seta only on femur, no setae on tibia	7
–	At least 2 setae on femur, 1 seta on tibia	8
7	4 basiconic sensilla on antennal article VI, 5 ornamental trichomes *c* to date found only in Australia	*Unixenus corticolus* sp. n.
–	3 basiconic sensilla on antennal article VI, 3 ornamental trichomes *c* to date found only in Ivory Coast, West Africa	*Unixenus vuillaumei*
8	3 basiconic sensilla on antennal article VI	9
–	4 basiconic sensilla on antennal article VI to date found only in NSW, Australia	*Unixenus barrabaensis* sp. n.
9	3 ornamental trichomes *c* per side, 2 pairs coxal glandsto date found only in Australia	*Unixenus mjoebergi*
–	5–9 (most commonly 8) ornamental trichomes *c*, 6 pairs coxal glands in male, to date found only in WA, Australia	*Unixenus karajinensis*

## Supplementary Material

XML Treatment for Unixenus

XML Treatment for Unixenus carnarvonensis

XML Treatment for Unixenus corringlensis

XML Treatment for Unixenus barrabaensis

XML Treatment for Unixenus myallensis

XML Treatment for Unixenus karajinensis

## Appendix

The record of all *Unixenus* specimens examined for this publication are available (doi: 10.3897/zookeys.278.4765.app). File format: Comma-separated values files (CSV).

## References

[B1] Burt J (1984) Report on the research of the pincushion millipede, *Unixenus mjoebergi* (Verhoeff, 1924) at Tom Price in the Pilbara of Western Australia. Western Australian Department of Agriculture, unpublished, 48 pp.

[B2] Condé B Jacquemin M (1962) Dipolopodes Pénicillates de Madagascar et des Mascareignes. Revue Française d’Entomologie 29 (4): 254-285

[B3] Condé B Terver D (1963) Pénicillates de Côte d’Ivoire (récoltes de M. Vuillaume). Bulletin Scientifique de l’Institut Fondamental d’Afrique Noire 25(A): 669–684.

[B4] Condé B Nguyen Duy-Jacquemin M (1984) Diplopodes Pénicillates de Papouasie et de Bornéo. Revue Suisse Zoologie 91: 47-55

[B5] Condé B Nguyen Duy-Jacquemin (2008) Classification actuelle des Diplopodes Pénicillates (Myriapodes) avec nouvelles définitions des taxa. Bulletin de la Société zoologique de France 133 (4): 291-302

[B6] Jones S (1937) On two new south Indian pselaphognathous diplopods. Zoologischer Anzeiger 119: 138-146

[B7] Jones S (1944) Mechanism of defence in a pselaphognathous diplopod, *Unixenus padmanabhii* Jones. Proceedings of the Indian Science Congress 31 (3): 94-95

[B8] Koch LE (1985) Pincushion millipedes (Diplopoda: Polyxenida): Their aggregations and identity in Western Australia. The Western Australian Naturalist 16(2/3): 30–32.

[B9] Manton SM (1956) The Evolution of Arthropodan Locomotory Mechanisms – Part 5: The Structure, Habits and Evolution of the Pselaphognatha (Diplopoda). Journal of the Linnean Society London 43: 153-187 doi: 10.1111/j.1096-3642.1957.tb02516.x

[B10] Nguyen Duy-Jacquemin M Condé B (1967)Mitteilungen aus dem Hamburgischen Zoologischen Museum und Institut 64: 43-81

[B11] Nguyen Duy-Jacquemin M Condé B (1982) Lophoproctidés insulaire de l’océan Pacifique (Diplopodes Pénicillates). Bulletin du Muséum d’Histoire Naturelle de Paris, 4e Série, Section A (1-2): 95–118.

[B12] Short M Huynh C (2011) The genus *Unixenus* Jones, 1944(Diplopoda, Penicillata, Polyxenida) in Australia. Zookeys 156: 105-122 doi: 10.3897/zookeys.156.2168 22303098 10.3897/zookeys.156.2168 PMC3253574

[B13] Silvestri F (1948) Tavola sinottica dei generi dei Diplopoda Penicillata. Bollettino del Laboratorio di Entomologia Agraria, Portici 8: 214-220

[B14] Verhoeff KW (1924) Results of Dr. E. Mjöbergi’s Swedish Scientific Expeditions to Australia 1910–1913. 34. Myriapoda: Diplopoda. Arkiv för Zoologi 16 (5): 1-142

