# Corrigenda: Short M, Huynh C (2013) Four new species of *Unixenus* Jones, 1944 (Diplopoda, Penicillata, Polyxenida) from Australia. ZooKeys 278: 75–90, doi: 10.3897/zookeys.278.4765

**DOI:** 10.3897/zookeys.291.5284

**Published:** 2013-04-17

**Authors:** Megan Short, Cuong Huynh

**Affiliations:** 1 Deakin University, 221 Burwood Highway, Burwood, Melbourne, Australia

When describing the new species *Unixenus corringlensis* Short & Huynh, sp. n., the latitude given for the single collecting event of this species in Corringle state forest is incorrect and should be 33°40'00"S not 33°22'12"S.

The correct wording in the species description for the holotype, a male paratype and two specimens included as other material is as follows:

Holotype. Female, Corringle State Forest, NSW, 33°40'00"S, 147°15'00"E (±1 km) (incorrectly recorded on label and in AM database as 33°22'12"S, 147°15'00"E, new latitude estimated with google earth), 21–26 March 1996, collected by D. Smith, AM KS.119541. Specimen mounted on slides,deposited in AM.

Paratypes. 1 male, same collection as holotype, AM KS.119540.

Other material. 2 immature specimens, (sex and stadia not determined), same collection as holotype, AM KS.53604, preserved in ethanol.

The Table of localities included as an appendix also requires the same correction. Portion of the table is shown below with corrections highlighted in yellow.

**Table:** Table of localities- part only, showing all entries for *Unixenus corringlensis* sp. n. Corrections highlighted in yellow.

Repository	Registration	Type status	Genus	Species	Specimens	Location	State	LatDS	LatMS	LatSS	LongDE	LongME	LongSE	Lat/long_source
AM	KS.119541	holotype	Unixenus	corringlensis	1F	Corringle State forest	NSW	33	40	00	147	15	0	Google earth estimate
AM	KS.119540	paratype	Unixenus	corringlensis	1M	Corringle State forest	NSW	33	40	00	147	15	0	Google earth estimate
AM	KS.87406	paratype	Unixenus	corringlensis	1M	Severn State forest, Atholwood Loop Rd	NSW	29	4	28	151	0	53	AM database
AM	KS.119542	paratype	Unixenus	corringlensis	1F	Severn State forest, Atholwood Loop Rd	NSW	29	4	28	151	0	53	AM database
AM	KS.119543	paratype	Unixenus	corringlensis	1F	Newnes State forest, Birds Rock Flora reserve, 0.6 km from Sunnyside Ridge Rd	NSW	33	19	43	150	11	23	AM database
AM	KS.87400		Unixenus	corringlensis	4	Severn State forest, Atholwood Loop Rd	NSW	29	4	28	151	0	53	AM database
AM	KS.87410		Unixenus	corringlensis	2	Severn State forest, Atholwood Loop Rd	NSW	29	4	28	151	0	53	AM database
AM	KS.53604		Unixenus	corringlensis	2 imm.	Corringle State forest	NSW	33	40	00	147	15	0	Google earth estimate
AM	KS.119544		Unixenus	corringlensis	1M, stadium VII	Newnes State forest, Birds Rock Flora reserve, 0.6 km from Sunnyside Ridge Rd	NSW	33	19	43	150	11	23	AM database

